# From the Soft to the Hard: Changes in Microchemistry During Cell Wall Maturation of Walnut Shells

**DOI:** 10.3389/fpls.2020.00466

**Published:** 2020-04-21

**Authors:** Nannan Xiao, Peter Bock, Sebastian J. Antreich, Yannick Marc Staedler, Jürg Schönenberger, Notburga Gierlinger

**Affiliations:** ^1^Institute of Biophysics, Department of Nanobiotechnology, University of Natural Resources and Life Sciences, Vienna, Austria; ^2^Division of Structural and Functional Botany, Department of Botany and Biodiversity Research, University of Vienna, Vienna, Austria

**Keywords:** cell wall, pectin, lignification, FTIR microscopic imaging, Confocal Raman microscopy, microchemistry, sclerenchyma, *Juglans regia*

## Abstract

The walnut shell is a hard and protective layer that provides an essential barrier between the seed and its environment. The shell is based on only one unit cell type: the polylobate sclerenchyma cell. For a better understanding of the interlocked walnut shell tissue, we investigate the structural and compositional changes during the development of the shell from the soft to the hard state. Structural changes at the macro level are explored by X-ray tomography and on the cell and cell wall level various microscopic techniques are applied. Walnut shell development takes place beneath the outer green husk, which protects and delivers components during the development of the walnut. The cells toward this outer green husk have the thickest and most lignified cell walls. With maturation secondary cell wall thickening takes place and the amount of all cell wall components (cellulose, hemicelluloses and especially lignin) is increased as revealed by FTIR microscopy. Focusing on the cell wall level, Raman imaging showed that lignin is deposited first into the pectin network between the cells and cell corners, at the very beginning of secondary cell wall formation. Furthermore, Raman imaging of fluorescence visualized numerous pits as a network of channels, connecting all the interlocked polylobate walnut shells. In the final mature stage, fluorescence increased throughout the cell wall and a fluorescent layer was detected toward the lumen in the inner part. This accumulation of aromatic components is reminiscent of heartwood formation of trees and is suggested to improve protection properties of the mature walnut shell. Understanding the walnut shell and its development will inspire biomimetic material design and packaging concepts, but is also important for waste valorization, considering that walnuts are the most widespread tree nuts in the world.

## Introduction

The nut is commonly defined as a dry, indehiscent, usually one-seed fruit with a hard and tough endocarp (shell) enclosing the seed, which develops from a simple ovary. The hardened endocarp of the nut provides a physical barrier around the seed and protects the embryo against biotic and abiotic factors in the natural environment (Dardick and Callahan, 2014). This remarkable design can be attributed to natural selection in the course of evolution (Sallon et al., 2008).

The walnut (*Juglans regia L*.), also known as English or Persian walnut, is the most widespread tree nut in the world, and is thus an economically important tree species (De Rigo et al., 2016). World production of walnuts exceeds three million tons since 2012 and took over almond and hazelnut (Bernard et al., 2018). Studies on nuts have proven their nutritional importance (Martinez et al., 2010), through antioxidant activities (Jahanbani et al., 2016; Panth et al., 2016). In a sustainable economy we furthermore can benefit from an optimized utilization of the nut waste—the shell and the husk—and for this, in-depth knowledge of these materials is of vital importance.

The rapid progress of genome sequencing of *Juglans* species was reviewed recently and will pave the way for functional genomics research (Chen et al., 2019). Fruit growth and development are a major research interest (Pinney and Polito, 1983; Wu et al., 2009) as well as the composition and nutritional value of the seed (Fukuda et al., 2003; Kornsteiner et al., 2006; Zhang et al., 2009; Martinez et al., 2010). In recent years, the walnut shell has also been studied due to its potential for the production of bioethanol (Yang et al., 2015; Lancefield et al., 2017), pyroligneous acid (Jahanban-Esfahlan and Amarowicz, 2018), charcoal and activated carbon (Xie et al., 2013) and “nutty carbon,” which is used for Na-ion battery anodes (Wahid et al., 2017). For novel applications and material development, fundamental knowledge of the structure as well as the chemistry of the shell is needed. Recently, a polylobate cell shape with interlocked packing was found to enable the superior mechanical properties of walnut shells (Antreich et al., 2019). All cells are connected via numerous pits (Reis et al., 1992; Antreich et al., 2019), which maintain symplastic connection by a recess of the cell wall (Reis et al., 1992). Chemical studies of the cell wall have shown that lignin is a main component (above 50%), followed by cellulose (25%), and hemicelluloses (22%) (Demirbas, 2005). During the differentiation of the walnut shell, the lignin content of the endocarp increases gradually, as shown by chemical analysis (Zhao et al., 2016). However, measuring total chemical composition of cellulose, hemicelluloses and lignin content typically requires tissue disruption and pretreatment to separate it from the cell wall matrix (Chen et al., 2015; Lancefield et al., 2017; Shah et al., 2018). Histochemical staining gives information in context with the structure (Li et al., 2018), but often lacks sensitivity among chemically similar components (Simon et al., 2018). For advanced understanding of the distribution of cell wall substances in the nutshell in context with the microstructure we explore the feasibility of vibrational microspectroscopy and imaging.

Vibrational spectroscopy methods such as Fourier-transform infrared spectroscopy (FT-IR) and Raman spectroscopy are increasingly used for chemical analysis in plant research, because they are fast, noninvasive, nondestructive, and require only limited sample preparation (Felten et al., 2015; Gierlinger, 2018). The two techniques often provide complementary information about the molecular vibrations of a given sample due to different energy transfers and thus different selection rules (Smith and Dent, 2005). FT-IR microspectroscopic imaging combines imaging with spectral information in a spatial context, which can provide an overview of all major chemical components of cell walls with a spatial resolution of about 10 μm (Mccann and Carpita, 2008; Mazurek et al., 2013; Yang et al., 2018). For chemical imaging with high spatial resolution Raman microscopy has shown a high potential via the selective acquisition of spectra from different cell wall regions at the sub-micron level (250 nm) (Gierlinger and Schwanninger, 2006, 2007; Gierlinger et al., 2012). Advances in laser technology, filter efficiency, CCD sensitivity and superior optics currently enable instruments to record signal with a good signal/noise ratio, which, in turn, paved the way for fast and sensitive scanning (Agarwal, 2006; Gierlinger et al., 2012). A Raman image is composed of thousands of pixels, and a complete spectrum can be acquired from each pixel. By selecting spectral positions which are unique to an individual chemical component, their spatial distribution can be visualized by an intensity heat map (Gierlinger, 2018). Microspectroscopy tools are thus powerful for characterizing dynamic chemical changes in the cell wall during development and maturation of fruits and vegetables (Chylinska et al., 2017).

The aim of this study is to understand the structure and composition of the mature walnut shell and its development. Analyzing chemical changes of nutshells in the context of microstructure during maturation (lignification) by FT-IR and Confocal Raman Microscopy will provide valuable insights into composition of the interlocked polylobate walnut cells and their “fabrication.” These results will inspire biomimetic material developments and promote the utilization of walnut shells for new products.

## Materials and Methods

### Plant Materials

Walnut (*Juglans regia*, cultivar “Geisenheim”) fruits were collected from various positions on the same tree, growing in the “BOKU horticulture Jedlersdorf” in Vienna, Austria once every month from June to October in 2017. At each developmental stage, ten similar sized fruits were selected, harvested and instantly stored at −20°C in plastic bags.

### Sample Preparation

Hand-cut equatorial sections of fruits from five developmental stages were cut with a razor blade and a saw. Then, 8μm-thick consecutive sections from July and October samples were cut with a cryostat microtome (CM3050S, Leica Biosystems Nussloch GmbH, Wetzlar, Germany) and rotary microtome (RM2235, Leica Biosystems Nussloch GmbH, Wetzlar, Germany), respectively. For FT-IR measurements, the sections were transferred onto a standard glass slide and dried in a desiccator for at least 48 h, then placed between two CaF_2_ windows (22 mm diameter, 0.5 mm thick) for FT-IR micro-spectroscopic imaging. The remaining consecutive sections were placed on a standard glass slide with a drop of distilled water, covered with a glass coverslip (0.17 mm) and sealed with nail polish. To test whether extractives are the reason for autofluorescence of the October samples the sections were treated with distilled water and 50% ethanol at 70°C for 48 h, respectively (as suggested by Queirós et al., 2019) and then measured by Confocal Raman Microscopy.

### Micro-Computed Tomography (Micro-CT)

Frozen walnuts from June to October (whole nuts with green husk) were scanned in an X-ray micro-computed tomograph (MicroXCT-200, Zeiss Carl Zeiss, Jena, Germany). Samples were put in a double-walled glass container and covered with Parafilm to keep the temperature low during the scan. Fast scans were performed (exposure time 1.0–1.3 s with binning 4) to minimize the artifacts from heating during the scan. Scanning parameters were set to 50 kV tube voltage and 160 μA current for the June to September samples, 40 kV and 200 μA for the October samples. Each scan consisted of ~500 2D radiographs with a voxel size of 89–100 μm3, depending on the total size of the sample. 3D data construction was performed via the software XMReconstructor 8.1.6599 (Zeiss Carl Zeiss, Jena, Germany).

### Histological Analysis

For histochemical staining, phloroglucinol (Wiesner) staining was performed as described in Yeung (1998). Briefly, hand-cut equatorial sections of five developmental stages were left for 20 min in phloroglucinol-HCl staining solution [20 mg/mL phloroglucinol in 20% ethanol and mixed with 12 N HCl (v:v=80:20)], and immediately photographed with a Canon EOS M10 fitted with a macro lens (35 mm, f/2.8 Canon Inc., Tokyo, Japan). The Wiesner reagent (phloroglucinol-HCl) mainly reacts with O-4-linked coniferyl and sinapyl aldehydes in lignifying cell walls (Pomar et al., 2002) and was used to follow the onset of lignification.

Sections from July and October nuts were stained with Fuchsin-Chrysoidine-Astrablue (FCA) solution [0.1 mg/mL of New Fuchsin, 0.143 mg/mL Chrysoidine, 1.25 mg/mL Astra blue and acetic acid (v:v=1:50)], and then washed step by step with distilled water, ethanol (30, 70%) and isopropanol. The samples were immersed in the FCA-solution for 48 h to guarantee a full penetration of the staining into the dense microstructure of the cell walls. Stained sections were embedded in Euparal and photographed under a Labophot-2 microscope (Nikon Corporation, Tokyo, Japan).

### Fourier-Transform Infrared (FTIR) Spectroscopy

A Vertex 70 Fourier-transform infrared (FT-IR) spectrometer, coupled to a Hyperion 2000 microscope (15× objective), which was equipped with a liquid nitrogen-cooled MCT-D316-025 (mercury cadmium telluride) detector and KBr beam-splitter (Bruker Optik GmbH, Ettlingen, Germany), was used to perform FT-IR mapping with an automated XY motorized stage. Both visible and spectroscopic imaging data of the sections were acquired at room temperature in transmission mode over the range of 4,000–700 cm^−1^ at an aperture size of 25 × 25 μm. Absorbance spectra were acquired at a spectral resolution of 8 cm^−1^. A rectangle area was selected to collect the FT-IR images of July (250 × 1500 μm) and October (300 × 1950 μm) samples, covering outer to inner tissues. A background spectrum of the CaF_2_ surface was collected before measuring the samples. The Opus 6.5 software (Bruker Optik GmbH, Ettlingen, Germany) enabled control of the microscope and collection of the spectra from the samples. Spectral data processing and image acquisition was performed using ImageLab (EPINA GmbH, Pressbaum, Austria) software, and the chemical mapping was displayed based on the intensity of functional groups. Then all the spectra were truncated to the finger print region (1,800–800 cm^−1^), baseline corrected and normalized using Opus 7.5 software (Bruker Optik GmbH, Ettlingen, Germany). Principal Component Analysis (PCA) was performed by Unscrambler X 10.3 software (CAMO Software AS, Oslo, Norway).

### Confocal Raman Microscopy

Raman spectra were acquired from microsections using a confocal Raman microscope (alpha300RA, WITec Ulm, Germany) equipped with a 100× oil immersion objective (NA 1.4, Carl Zeiss, Jena, Germany) and a motorized XYZ stage. A linear polarized (0°) coherent compass sapphire green laser (λ_ex_ = 532 nm, WITec, Ulm, Germany) was passed through a polarization-preserving single-mode optical fiber and focused through the objective with a spatial resolution of 0.3 μm on the sample. The Raman scattering signal passed a multi-mode fiber (50 μm diameter) and was detected by a CCD camera (Andor DV401 BV, Belfast, UK) behind a spectrometer (600 g mm^−1^ grating, UHTS 300 WITec). The laser power used on the July and October section was 34.7 and 10 mW, respectively. The sample was mapped by collecting single spectra at every image pixel, with an integration time of 0.04 s per spectrum, a spectral resolution of 4 cm^−1^ in the range of 3,800–300 cm^−1^. The monochromator of the spectrometer was calibrated using the Raman scattering line produced by a silicon plate (520 cm^−1^). For measurement setup the Control Four (WITec, Ulm, Germany) was used.

Raman data analysis was performed with Project Four (WITec, Ulm, Germany) and Opus 7.5 software (Bruker Optik GmbH, Ettlingen, Germany). After applying cosmic ray spike removal, Raman chemical images were generated based on the integration of relevant wavenumber regions (e.g., CH stretching, lignin, cellulose, pectin, and fluorescence), which enabled definition of cell wall areas of interest and calculation of average spectra from these regions. All calculated spectra were exported into Opus 7.5 for comparison and baseline correction.

## Results

### Morphological Analysis of Shell Development

**Figure 1:**
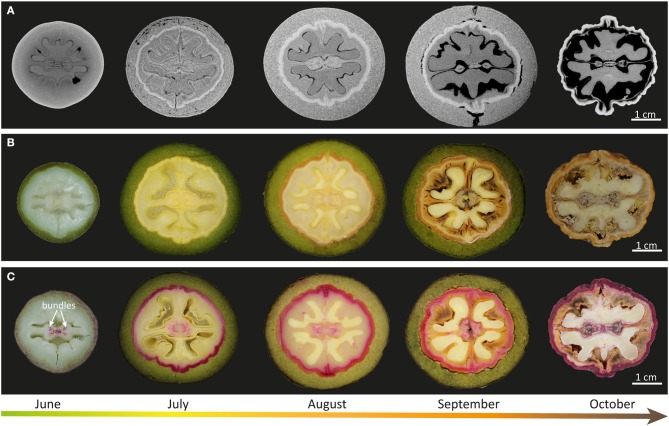
Shell morphology of walnuts at different developmental stages. **(A)** Micro-computed tomography (micro-CT) images of the horizontal plane of walnuts harvested from June to October (from left to right). **(B)** Representative pictures of equatorial sections of walnuts fruits in the native state and **(C)** after staining with 1% phloroglucinol-HCl.

Walnut fruits completed their development and ripening within six months (May-October 2017) (Figure 1). The spatial organization of the walnut at five developmental stages was visualized in 3D scans (Supplementary Videos 1–5) based on micro-CT data. The reconstructed 3D models of the walnut fruit allow for the dissection of the tissues into 2D virtual sections (Figure 1A). Due to differences in tissue density the developing seed and the different shell layers can be visualized in the different stages. Dense tissues appear light, while dark areas represent air space (Verboven et al., 2012). In June the outer layer appeared homogenous, but in July a distinct light concentric layer between the outer layer and the seed was apparent (see Figure 1A). This layer with higher density represented the onset of nutshell differentiation. The shell region became thicker during tissue maturation and formed a seal around the seed. Upon maturation (in September) more and more air (dark regions) was found around the seed and also between the shell and the green outer husk, which finally was shed in October (Figures 1A,B). In the photographic images of the equatorial sections, the onset of shell differentiation was apparent as a light yellow layer (Figure 1B). During maturation the shell became brown: first close to the green husk (August) and later uniformly brown (October) (Figure 1B). The onset of lignification based on Wiesner staining was detected close to the green husk as a pink layer (Figure 1C). In August, this concentric tissue layer underlying the green husk became more red stained, while the inner part was still pink. The strong lignification of the shell was clearly visible until the final stage in October, although the shades of red changed during development (Figure 1C).

**Figure 2:**
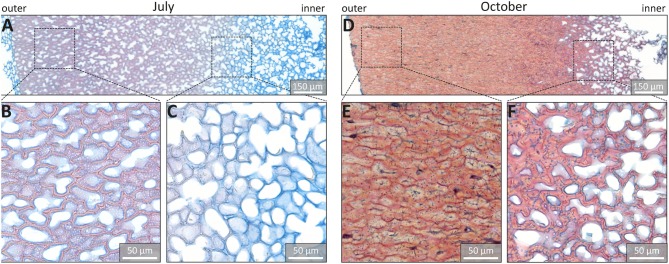
Histological staining (FCA) of microsections from walnut shells. **(A,D)** Overview of sections of walnut shell sampled in July and October, respectively. **(B,C)** details of **(A)** and **(E,F)** details of **(D)** viewed by bright-field light microscopy.

Fuchsin-Chrysoidin-Astra (FCA) staining of microsections confirmed that lignification began in July in the outer part of the shell (Figures 2A,B), while the very thin walls of the inner part were stained only blue and thus not yet lignified (Figures 2A,C). The October sample was stained even more reddish and the cell wall thickness increased, especially in the outer part of the shell (Figures 2D,E). In the very inner part the cell walls were thin, but stained red and were thus also lignified (Figure 2F).

### FT-IR Imaging of Walnuts Shell Micro-Sections

**Figure 3:**
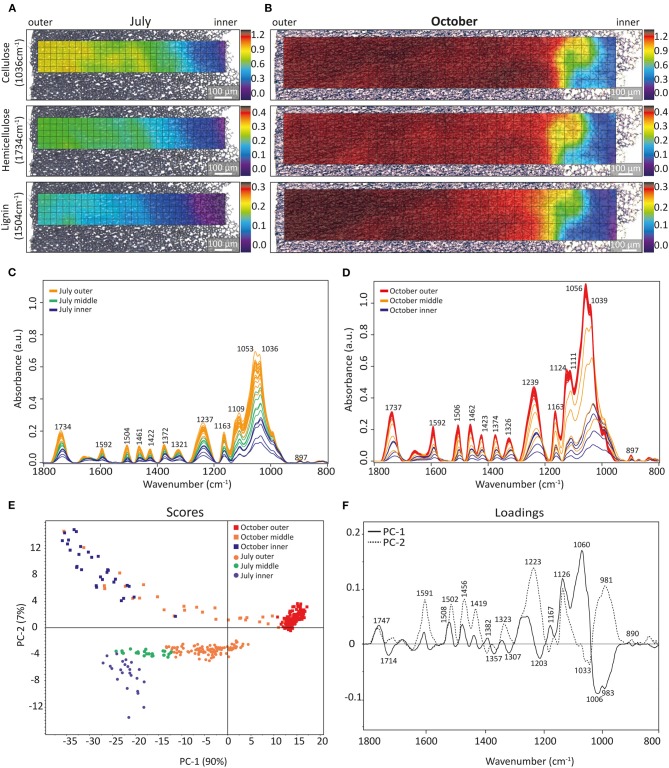
IR image reconstruction of sections of walnut shells from July and October. Chemical mapping overlaid with bright-field images of sections based on the integrated absorbance of cellulose (1,036 cm^−1^), hemicelluloses (1,734 cm^−1^), and lignin (1,504 cm^−1^) of a microsection from July **(A)** and October **(B)**. A rainbow scheme has been used to denote absorbance, with the warmest colors (red) indicating the highest absorbance, and cool colors (blue) representing a low spectral intensity. **(C,D)** Show comparison of baseline corrected spectra of the 1st line from outer to the inner part of sections from July and October, respectively. Score plots **(E)** of principal component analysis (PCA) of infrared spectra obtained from the walnut shell sections of July and October after baseline-correction and normalized at 1,374 cm^−1^ and the corresponding loadings **(F)** of PC1 and PC2.

Fourier-Transform Infrared (FT-IR) microscopy was applied to probe the chemical composition of the walnut shells based on the absorption at particular infrared light frequencies corresponding to specific chemical bonds and groups of the different cell wall polymers (Figure 3). The strongest bands between 1,015 and 1,060 cm^−1^ are assigned to C-O stretching vibrations of cellulose (Fengel and Ludwig, 1991); therefore, the intensity at 1036 cm^−1^ was used to monitor cellulose distribution (Figures 3A,B). For hemicelluloses the absorption band at 1,734 cm^−1^ is indicative and assigned to C=O stretching vibration of acetyl groups (Faix, 1991) and here used to follow the hemicelluloses. The band centered at 1,504 cm^−1^ is assigned to aromatic skeletal vibrations of lignin (Faix, 1991) and used for imaging the aromatic components in the shell. The intensity-based color-coded maps of the three cell wall polymers, cellulose, hemicelluloses, and lignin allowed us to visualize the changes across the shell (outer to inner part) for samples collected in July (Figure 3A) and October (Figure 3B). In the July sample (Figure 3A), the top map reveals that the highest cellulose band intensity (yellow) lies in the outer part of the shell (nine-fold higher than that of the inner part). The maps for hemicelluloses (1734 cm^−1^) and lignin (1,504 cm^−1^) showed a similar intensity pattern to those obtained for cellulose (Figure 3A). In contrast, in October maps, intensity distribution was homogenous over a large part of the measured area, with a steep gradient confined to the inner part (Figure 3B). The shape of the spectra of the outer and inner regions was similar for the 2 months, but the absolute intensities differed and the lignin marker bands (1,504, 1,592 cm^−1^) were not detected in the very inner part of the July sample (Figures 3C,D). The cellulose intensity in the October sample was roughly twice that of the July sample, whereas hemicelluloses increased by around 1.5 times and the lignin signal was around three times stronger in the October sample compared to the July sample (Figures 3C,D).

By normalizing FT-IR spectra on the 1,374 cm^−1^ band, the major intensity differences due to thickening of the cell wall are canceled out and compositional differences can be analyzed using principal component analysis (PCA) (Figures 3E,F). Composition along the section in the October sample is less variable than in the July sample as illustrated by the tight grouping of the outer part on the right side along the axis of Principal Component 1 (PC1, red squares). PC1 explains 90% of the spectral variability and spreads the samples from inner to outer part for the October and July samples along the PC1-axis. Spectra from the outer shell (orange round) and middle part (green round) of the July sample are along PC1 in the same region as the middle part of the October sample (Figure 3E, orange squares). The PC1-loading reveals bands typical for hemicelluloses (e.g., 1,747 cm^−1^), lignin (e.g., 1,508 cm^−1^) as well as cellulose (e.g., 1,167, 1,126, 1,060 cm^−1^) as observed in the walnut spectra (compare loading 1 Figure 3F with spectra of Figures 3C,D). However, some bands have proportionally lower intensities (1,591, 1,323, 1,239 cm^−1^); moreover a strong negative band at 1,006 cm^−1^ (shoulder 983 cm^−1^) as well as strong medium bands at 1,714, 1,357, 1,307, and 1,203 cm^−1^ are observed (Figure 3F, loading 1). PC2 explains another 7% of the variability and separates July samples from October samples and especially the spectra of the inner parts of the shell (Figure 3E, blue markers). The loading 2 has strong contributions at 1,591, 1,502, 1,456, 1,419, 1,323, 1,223, 1,126 cm^−1^.

### Raman Imaging

**Figure 4:**
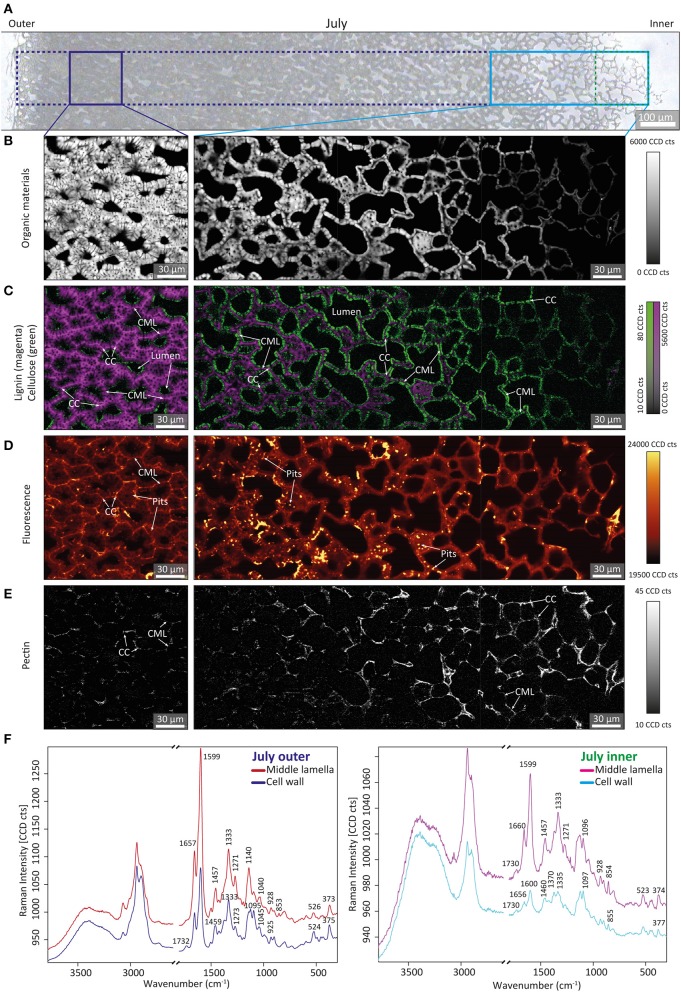
Bright field photomicrograph and representative Raman images of walnut shell section of July. **(A)** Bright field image of a microsection of walnut shell from outer to inner. **(B–E)** Raman images (150 × 150 μm) were calculated by integrating over **(B)** CH -stretching region 2,745–3,054 cm^−1^. **(C)** Lignin around the region 1,535–1,704 cm^−1^ (in magenta) overlaid with cellulose bands (1,358–1,401 cm^−1^, in green). **(D)** Fluorescence region. **(E)** Intensity of pectin (855 cm^−1^) band. **(F)** Average spectra extracted from the cell wall and compound middle lamella area of the outer (blue) and very inner part (green) of walnut shell section of July, respectively.

**Figure 5:**
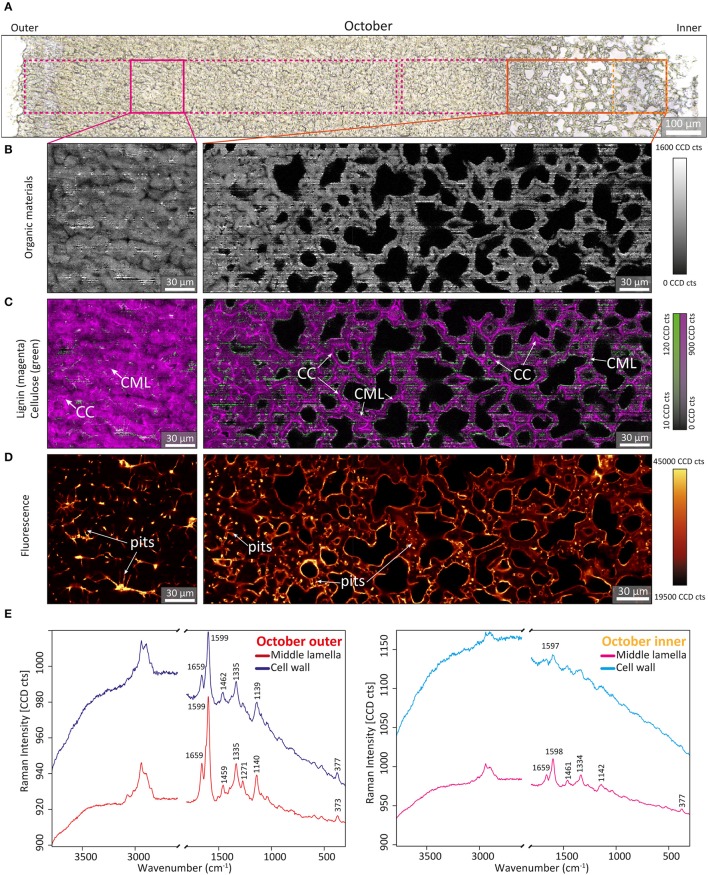
Bright field photomicrograph and Raman images of walnut shell section of October. **(A)** Bright field image of a microsection of walnut shell from outer to inner. **(B–E)** Raman images (150 × 150 μm) were calculated by integrating over **(B)** organic materials region 2,745–3,054 cm^−1^. **(C)** Lignin around the region 1,535–1,704 cm^−1^ (in magenta) overlaid with cellulose bands (1,358–1,401 cm^−1^, in green). **(D)** Fluorescence region. **(E)** Average spectra extracted from the cell wall and compound middle lamella area of the outer (purple) and very inner part (yellow) of walnut shell section of October, respectively.

In the next step, Confocal Raman microscopy was applied to obtain detailed insights into the molecular composition of the nutshell at the microscale. The measurement area covered again the entire shell from the outer to the inner part (Figures 4A, 5A and Supplementary Figures 1, 2). To highlight developmental changes, representative areas were selected: one region from the outer part of the shell and a wider region from the inner part, where most of the changes were observed by FT-IR (Figure 3). To get an overview of the microstructure, chemical images were generated by integrating over the CH-stretching region (2,745–3,054 cm^−1^), including signal contributions from all cell wall polymers (Gierlinger et al., 2012) (Figures 4B, 5B and Supplementary Figures 1A, 2A). In the July sample, highest CH-stretching intensity (light color) was found in the left image (representative for the outer part), and a substantial decrease in intensity toward the seed—the inner side of the shell (Figure 4B, Supplementary Figure 1A). In the contrast, in the section of the October sample (Figure 5B, Supplementary Figure 2A), an overall high intensity was observed in the outer as well as in the inner part.

The integral of the lignin-specific region 1,535–1,704 cm^−1^ (Gierlinger and Schwanninger, 2007) was combined with the integration of the 1,380 cm^−1^ cellulose band, which highlights non-lignified regions (Gierlinger, 2018). In these combination images areas without lignin are clearly visualized in green and at the same time the lignified regions are highlighted in magenta (Figures 4C, 5C). In the July sample, the highest intensity (magenta color) of the lignin band was observed in the outer part, while the cellulose integration (1,380 cm^−1^) highlighted the thin cell walls of the inner part (Figure 4C, Supplementary Figure 1B). At the micro-scale, a higher amount of aromatics was visualized in the cell corners (CC) and compound middle lamella (CML) compared to the cell wall. In the thicker cell walls, an inner, still not yet lignified layer (green) was detected, close to the lumen (Figure 4C, left image). In the October samples, aromatic components were visualized throughout the whole shell (in magenta; Figure 5C, Supplementary Figure 2B). Even in the inner part the thin walls were lignified and high amounts of aromatics were found in the CC and CML (Figure 5C, right side).

By plotting the overall intensity change of the background, which stands for all fluorescing components, additional details were visualized (Figures 4D, 5D, Supplementary Figures 1C, 2C). Especially in the zone where lignification begins (Figure 4C, magenta), highly fluorescing components were found in the pits, the numerous cell to cell connections and partly on the inner surfaces of the cells (Figure 4D, middle). In the October samples, the fluorescing layer toward the lumen is even more pronounced in the inner part (Figure 5D, right side). In the outer part of the July sample, CML and CC showed higher fluorescence and coincided with the lignin distribution (Figures 4C,D, left image). The outer part of the October sample showed the background with the highest fluorescence between the cells and inside pit channels (Figure 5D, left image).

Finally, pectin distribution was visualized by integrating over the 857 cm^−1^ band, assigned to pectin (Synytsya et al., 2003). In the July sample, pectin was clearly detected throughout the entire shell (Figure 4E). Unlike other cell wall components pectin increased in the inner part (Figure 4E, right side). The distribution on the micro-scale showed an accumulation in CC and CML, especially in the unlignified regions (Figure 4E, right side). The derived spectra from the cell wall and the middle lamella of the shell of the July sample (Figure 4F) clearly confirmed the pectin band at 855 cm^−1^ in the July samples, beside the characteristic bands for cellulose and lignin. The increase of the aromatic stretching vibration at 1,599 cm^−1^ from the inner (right side) to the outer shell part (left side) is clearly seen as well as the higher amount in the middle lamella compared to the cell wall (red vs blue spectra). In the October sample the pectin marker band could neither be observed in the inner part, nor in the outer part as confirmed in the derived average spectra from the cell corners and cell walls (Figure 5E). In all spectra lignin bands dominate (1,599, 1,335, 1,140 cm^−1^) and contrary to what was observed in the shell of the July sample (Figure 4F), the fluorescence background in the cell wall is much higher than in the middle lamella (Figure 5E). Extraction of the micro-sections (with the aim to remove extractable components) did not result in lower fluorescence background and better spectral quality (Supplementary Figure 4).

**Figure 6:**
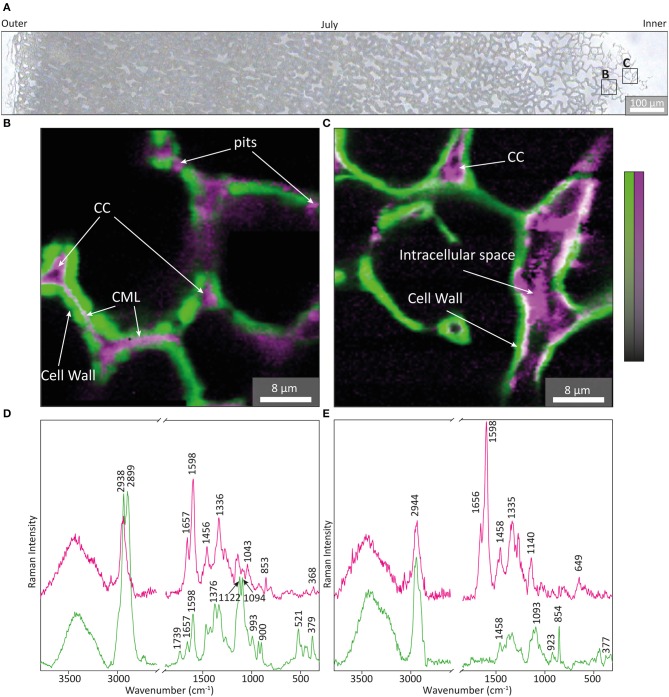
Non-negative matrix factorization (NMF) analysis of the inner July shell. **(A)** Bright field image of a microsection of walnut shell of July with the two positions used for analysis marked in the innermost right side. Colored composite images **(B,C)** showing the two representative endmember (EM) abundance maps of cell wall (green) and cell corner (magenta) and the corresponding endmember spectra **(D,E)** after baseline correction.

Zooming into the inner part of the July sample (Figure 6A) and applying a multivariate unmixing approach, the onset of lignification was visualized in detail (Figures 6B–E). Non negative matrix factorization (NMF) delivers the most pure component (endmember) spectra and their abundance maps (Prats-Mateu et al., 2018). One component was retrieved from CC, CML and pits (Figure 6B) and showed clear aromatic bands at 1,657, 1,598, 1,335, 1,140 cm^−1^ together with the pectin band at 853 cm^−1^ (Synytsya et al., 2003) (Figure 6D, magenta). The onset of cell wall thickening goes hand in hand with lignification as proven by the fact that the most pure component from the cell wall (Figure 6B, green), clearly included aromatic bands, although less intense (Figure 6D, green spectrum). In the innermost thinner walls the aromatic component was restricted to the cell corner and intracellular space (Figure 6C, magenta) and an additional band at 649 cm^−1^ was detected (Figure 6E, magenta). The endmember spectrum of the thin cell wall in the innermost part (Figure 6C) shows a strong band at 856 cm^−1^ [assigned to pectin (Synytsya et al., 2003)] beside cellulose bands [e.g., 1,093, 377 cm^−1^; (Wiley and Atalla, 1987)], but no aromatic band at 1,600 cm^−1^ (Figure 6E, green). In this innermost part of the July nutshell aromatic components are present not in the cell wall, but only in the space between the cells and cell corner (Figures 6C,E, magenta).

## Discussion

### Green Husk: Production and Protection of the Nutshell

X-ray tomography is a non-destructive method gaining popularity for internal quality evaluation in various fields of agriculture and food quality evaluation (Kotwaliwale et al., 2014). In this study, the technique gave a detailed overview of the structure of the whole fruit during development (Figure 1A and Supplementary Videos 1–5). In the June sample, the shell is not clearly distinguishable from the husk, only the bundles indicate the position. From the July to the September samples, the shell layer is clearly visible and increases in thickness, while around the seed more and more air space is arising (black area). Finally the much denser (brighter on the scans) and dry nutshell protects the seed, which is mostly surrounded by an empty cavity (Figure 1A, Supplementary Movie 5). Zooming into the nutshell via x-ray tomography showed that the entire shell is composed of polylobate cells, each of which tightly interlocked with on average 14 neighbors (Antreich et al., 2019). While other nutshells, like Macadamia build up their cells with different cell types, i.e., fibers and sclerenchyma cells (Schuler et al., 2014), walnut relies only on the puzzle cell type (Antreich et al., 2019). This explains why no layering within the walnut shell is visible by X-ray-tomography.

The drying process, especially during the last month of development is represented by a strong black contrast and results in a more and more “wrinkled” and dense (white) nutshell (Figure 1A). The outer husk, which protects the nut during the shell formation, is gray on the CT scans and is thus a less dense layer. During the first month until July it increases in thickness, but stays constant during shell formation. Also light-microscopic images before and after staining (Figures 1B,C) confirm the protecting husk to show major changes from June to July, but staying constant in terms of size and color during shell development (July-September). The color changes in the inner shell from light yellow to brown (Figure 1B) point to a change in lignin amount and/or impregnation with other phenolic substances. Staining the nut halves with Wiesner reagent, confirmed the presence of cell wall associated phenolic molecules (phenolic acids and C6-C1 derivatives of hydroxycinnamaldehydes/alcohols) in the nutshell by red coloration from July on (Figure 1C). Similar studies on Chinese walnut varieties (“Zanmei” and “Zhenzhuxiang”) showed the beginning of coloration and thus lignification on June 16th and reported the finished shell layer differentiation one month later (July 20th) (Zhao et al., 2016). Although the shell is finally the most stained region, the very first beginning of lignification in the June sample takes place in the very inner part of the diaphragm along the two major bundles supporting the kernel (Figure 1C, June). Being the first part to be impregnated with aromatics this walnut septum was recently proven as a rich source of polyphenols and has been used as a traditional nutraceutical material (Liu et al., 2019). Within the shell the first cells to become sclerified were reported to be those at the micropylar end, proceeding basi-petally with the cells along the suture lines becoming very rapidly sclerified (Pinney and Polito, 1983).

Cross-sections stained with FCA confirmed the start of lignification in July (Figure 2A). The outer part (toward the green husk (Figures 2A,B) is more reddish than the inner part (Figure 2C). This outer part is adjacent to the fleshy outer green husk, which provides nutrition for the seed's growth (Wu et al., 2009). Vascular bundles are found at the border in the surrounding husk and are probably involved in providing and transporting the needed resources for the development of the shell (Supplementary Figure 3), although they are not passing through the shell like in Macadamia (Schuler et al., 2014) or coconut (Schmier et al., 2000). The cell walls adjacent to the green husk are the first to be impregnated with aromatic components in the developing nutshell from July on (Figures 2A, 3A,C, 4C,F). The beginning of lignification in this outer part and the decrease in the amount of aromatics from the green husk toward the inner part points to the role of the green husk in providing components for maturation of the shell.

### Nut Shell: Secondary Cell Wall Formation and Lignification

Raman spectroscopy is a very suitable tool to detect the onset of lignification, because of the signal enhancement of conjugated aromatic compounds. Lignin monolignols—the main building blocks of lignin (Vanholme et al., 2010)—are such compounds and give rise to a prominent band at 1,600 cm^−1^. This band itself is only indicative for an aromatic ring stretch, but its relative intensity to other bands can be interpreted as caused by an aromatic ring participating in a conjugated π-system. With the co-appearance of the bands at 1,657, 1,333, 1,271, and 1,140 cm^−1^ the acquired Raman spectra can with good confidence be assigned to coniferyl alcohol and aldehyde (Bock and Gierlinger, 2019). Based on the signal enhancement of these monolignols the onset of lignification can be precisely monitored by following their distribution during cell wall formation at different developmental stages (Figures 4–6). Based on the spectra we can not distinguish if coniferyl alcohol and alcohol are present as monolignols in the developing cell wall or as endgroups in a continuously growing lignin polymer. The fact that neither extraction of young developmental stages nor the mature nut shells changed their aromatic spectral signature, points to detection of endgroups of the lignin polymer.

The inner part of the nutshell sampled in July represents thin primary cell walls, as proven by the derived plant cell wall endmember spectrum (Figure 6E, green spectrum) with characteristic marker bands of pectin at 856 cm^−1^ (Synytsya et al., 2003) and cellulose [e.g., 377, 1,093 cm^−1^, (Wiley and Atalla, 1987)] and the absence of the aromatic 1,600 cm^−1^ vibration. However, coniferyl alcohol and aldehyde bands are detected in the space between two cells, at the cell corner and at the lumen sided surfaces (Figure 6C). Two cells toward the green husk the cell thickness almost doubled (Figure 6B) and aromatic components spread clearly in the middle lamella as well as in the pits (Figure 6B), which seem to be pathways to transport the monolignols. The numerous pits found in stone cells are reported to maintain symplastic connection between cells by a recess of the cell wall (Reis et al., 1992; Sebaa and Harche, 2014). When the middle lamella is filled up with monolignols, they spread into the secondary cell wall as proven by the aromatic bands in the endmember spectra of the cell wall (Figure 6D, green spectrum). How lignin monomers are trafficked from inside the cell to the cell wall is currently debated and possible mechanisms involve transporters, the diffusion of monomers across lipid bilayers and the release of monolignol glucosides stored in vacuoles (Perkins et al., 2019). For the nutshell we could show a clear tracking of monolignols from lumen over pits to first accumulate in the middle lamella and cell corners between the cells, before impregnating the cell wall. In the state of primary cell wall (Figures 6C,E) the monolignol dominated endmember spectrum shows an additional band at 648 cm^−1^, which might be attributed to proteins involved in monolignol synthesis. There is not yet a comprehensive model of the mechanisms of monolignol export and above reported mechanisms are not mutually exclusive, but may predominate in different tissues and in different developmental stages (Perkins et al., 2019).

Another interesting fact is the clear Raman detection of pectin within the primary cell wall on the innermost part of the shell (Figures 6C,E, green) and filling together with lignin the space between the cells (CC, CML, and pits in Figures 6B,D magenta) (Figure 4E). Raman imaging shows a clear decrease in intensity of the pectin band (853 cm^−1^) toward the green husk in the nutshell sampled in July (Figure 4E), while at the same time the aromatic 1600 cm^−1^ stretching band increased (Figure 4C). By microautoradiography of polygalcturonan deposition it was shown that pectin formation terminated when secondary cell wall synthesis began (Imai and Terashima, 1992). Using pectin sensitive antibodies the absence of pectin in the secondary cell wall has been verified in pine xylem (Hafren et al., 2000) and stone cells of Norway spruce phloem (Kim and Daniel, 2017). In a very recent study on xylem cell wall formation in pioneer roots and stems of poplar pectins did not co-localize in lignified cell walls, but were found in primary tissues (Marzec-Schmidt et al., 2019). The role of acidic pectin in secondary cell wall formation of the nutshell is confirmed by our intense pectin band at 853–854 cm^−1^ in the July sample (Figures 4F, 6D,E), which corresponds exactly to the same position as Polygalacturonic acid (Synytsya et al., 2003). This band was found to monitor changes in pectin composition, as it decreases with methylation (min. 850 cm^−1^) and increases with acetylation (max. 862 cm^−1^) (Synytsya et al., 2003). Although the July sample includes thin unlignified primary cell wall toward the seed (Figures 6C,E) and thicker lignified cell wall toward the green husk (Figure 4D), the pectin composition based on the Raman band position stays constant while the amount changes (Figures 4F, 6D,E). These results in the nutshell development coincide with the observation that pectin is partly degraded on maturation of secondary cell wall formation in wood (Westermark, 1982; Westermark et al., 1986) and induces lignification (Robertsen, 1986).

### Maturation of the Shell by Impregnation and Dehydration

The color changes from July to October in the native (Figure 1B) as well as the stained nut halves (Figure 1C) indicate an ongoing nut shell maturation in the last three month of walnut development. The thicker cell walls in October are even more reddish, suggesting higher amount of lignin and/or additional impregnation with extractives (Figures 2B,E,F).

The score plot of the PCA analysis of the FT-IR spectra (Figure 3E) confirmed spectral and thus chemical differences between July and October sample and across the tissue from the inner (toward seed) to the outer part (toward green husk). Along the PC1 axis (explaining 90% of the variability), most of the spectra of the October sample built a cluster on the positive right side (Figure 3E, red circles), while the spectra from the outer position of the July sample fall together with the spectra of the middle part of the October sample (Figure 3E). Some bands in the PC1 loading (Figure 3F) point to a change in aromatic components (e.g., aromatic skeletal vibration 1,508 cm^−1^, C-H deformation combined with 1,456 cm^−1^, reported to be common in different lignin samples (Boeriu et al., 2004). Another remarkable band increasing in October outer is the 1,126 cm^−1^ band, reported to be characteristic for S-lignin (Cai et al., 2010), but might together with the 1,060 cm^−1^ band, also come from contributions of tannins (Arshad et al., 1969). PC2 explains another 7% and separates July from October samples and especially the spectra of the inner parts of the shell (blue markers, Figure 3E). The loading 2 has strong contributions at 1,591, 1,502, 1,456, 1,419, 1,323, 1,223, and 1,126 cm^−1^, all typical bands in S-lignin (Faix, 1991) and/or together with 981 cm^−1^ interpreted as tannins (Arshad et al., 1969). A high lignin content around 45% was reported for walnut shell and considered to be SGH type with dominant G-units (86.7%) (Li et al., 2018), while others come up with 30% lignin content with an S/G ratio of 1.6 (Queirós et al., 2019). Extractive content was reported 7–10% (Li et al., 2018; Queirós et al., 2019) with a high amount of phenolics, including flavonoids and tannins (Queirós et al., 2019). Recently hydroxystilbenes, a class of nonflavonoid polyphenolics, have been found to be part of the lignin structure of palm fruit endocarps based on nuclear magnetic resonance spectroscopy (Carlos Del Rio et al., 2017; Rencoret et al., 2018). The incorporation of piceatannol into the lignin polymer was suggested to have a role in seed protection (Rencoret et al., 2018).

Raman microscopy showed the effect of maturation by a tremendously increasing fluorescence background (compare Figures 4, 5). Unfortunately, the higher background of the Raman spectra only provides information on the strongest aromatic stretching vibrations, whereas at some places, especially the pits and the lumen, no bands could be resolved. Raman imaging results suggest a final impregnation of the tissue through the numerous pit channels by additional aromatic components as the fluorescence signal is increasing in the cell wall, but even more in the pits (Figure 5D and Supplementary Figure 2C). Extraction of the sections did not remove the fluorescence background (Supplementary Figure 4), which points to a non-extractable phenolic component or lignin. However, lignin autofluorescence imaging showed the highest intensity in the cell corner and middle lamella (Supplementary Figure 5A) in contrast to the Raman fluorescence lining the cells and the pits (Supplementary Figure 4). Together with the SEM images (Supplementary Figure 5B), which shows components sticking to the wall, we therefore conclude that aromatic components other than lignin stick to the inner wall of the cells and seal the pits (Figure 5D). Although not soluble by the usual extraction procedure additional aromatics are impregnating the walnut tissue in the mature state. Raman imaging of pine wood showed a similar accumulation of aromatic components (pinosylvins) during heartwood formation - with lumen sided surfaces and pits more impregnated than the cell wall itself (Felhofer et al., 2018). This final impregnation step of the cell wall, filling up free spaces, and sealing the pit channels will improve stability and longevity in a similar manner as observed in heartwood of trees.

Although wood and nutshell have different mechanical functions, which are realized by isotropic puzzle cells (Antreich et al., 2019) for high compression forces and anisotropic fiber arrangement for high tensile strength respectively (Gibson, 2012), the underlying secondary cell wall and its maturation show common features.

## Data Availability Statement

The datasets generated for this study are available on request to the corresponding author.

## Author Contributions

NG and NX conceived the experimental design and data analysis. NX and SA performed light microscopy and SEM microscopy. NX and PB conducted the FTIR and Raman spectroscopy. SA, YS, and JS contributed in design of the micro-CT experiment and in the analysis of the data. All authors contributed in writing and reviewing the manuscript.

## Conflict of Interest

The authors declare that the research was conducted in the absence of any commercial or financial relationships that could be construed as a potential conflict of interest.

## Supplementary Material

Supplementary Video 1 Micro-CT video of walnut fruits harvested from June.

Supplementary Video 2 Micro-CT video of walnut fruits harvested from July.

Supplementary Video 3 Micro-CT video of walnut fruits harvested from August.

Supplementary Video 4 Micro-CT video of walnut fruits harvested from September.

Supplementary Video 5 Micro-CT video of walnut fruits harvested from October.
