# Supplementary material for: From the Soft to the Hard: Changes in Microchemistry During Cell Wall Maturation of Walnut Shells
Source: Front Plant Sci. 2020 Apr 21;11:466. doi: 10.3389/fpls.2020.00466 (PMC7216782; doi:10.3389/fpls.2020.00466)
Supplement: Supplementary file 6 [file Data_Sheet_1.PDF]

Supplementary Material

Supplementary Videos

July_movie.avi June_movie.avi August_movie.avi October_movie.avi September_movie.avi

Supplementary Figures

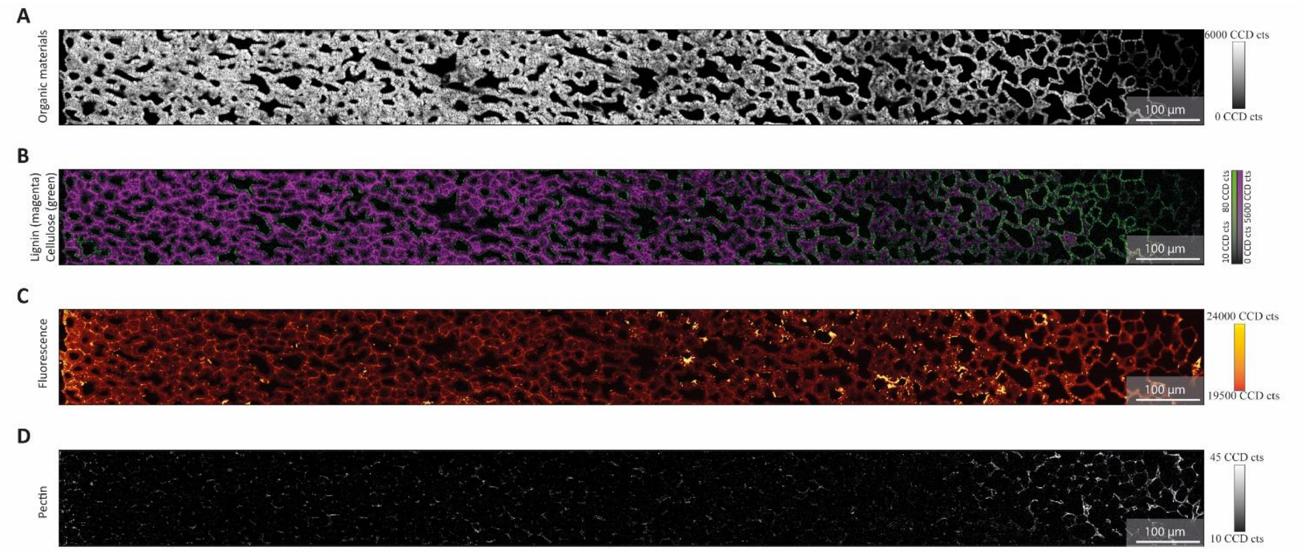

Supplementary Figure 1. Representative Raman images of the whole walnut shell section of July. (A-F) Raman images (1800 µm × 150 µm) were calculated by integrating over (A) CH-stretching region 2745-3054 cm^{-1}. (B) Lignin around the region 1535-1704 cm^{-1} (in magenta) overlaid with cellulose bands (1358-1401 cm^{-1}, in green). (C) Fluorescence region (D) Pectin around the region (839 cm-1-872 cm^{-1}).

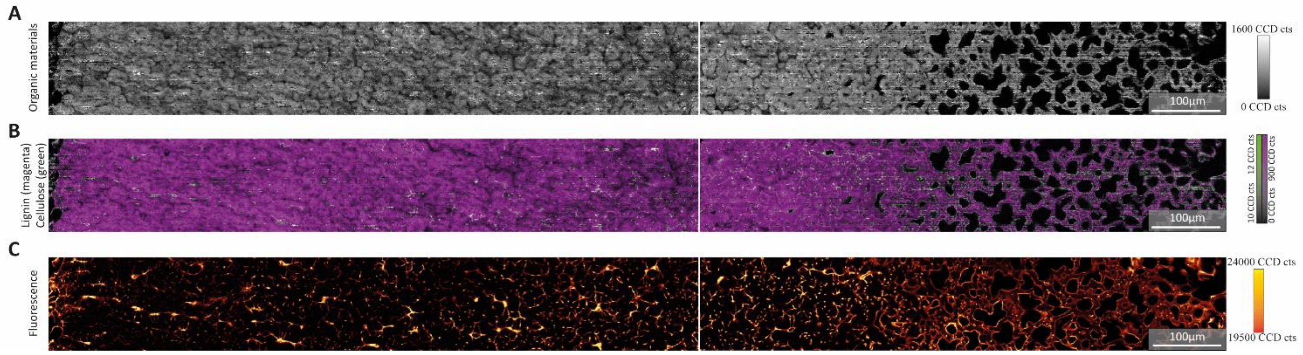

Supplementary Figure 2. Representative Raman images of the whole walnut shell section of October. (A-E) Raman images (1800 µm × 150 µm) were calculated by integrating over (A) CH-stretching region 2745-3054 cm^{-1}. (B) Lignin around the region 1535-1704 cm^{-1} (in magenta) overlaid with cellulose bands (1358-1401 cm^{-1}, in green). (C) Fluorescence region.

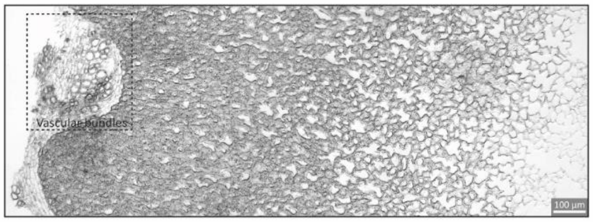

Supplementary Figure 3. Bright field photomicrograph of walnut shell section of July, showing the vascular bundles in the adjacent green husk.

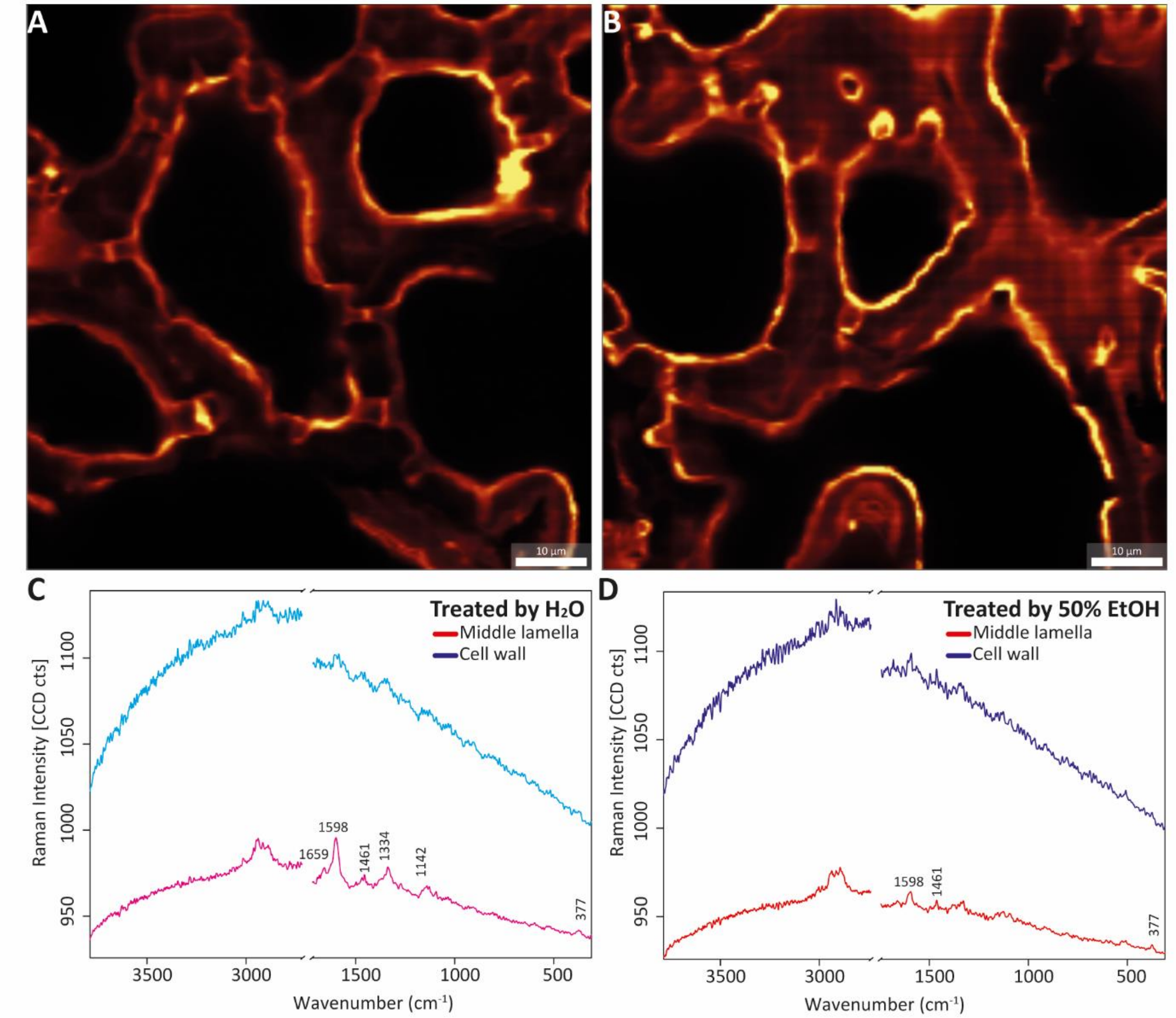

Supplementary Figure 4. Comparison of Raman images of walnut shell inner part from October treated by H2O and 50% Ethanol, respectively. (A and B) Raman images based on integrating over fluorescence region, corresponding to treatment of H2O and 50% EtOH at 70 °C for 48h, respectively. (C and D) Average spectra extracted from the cell wall and compound middle lamella area, respectively.

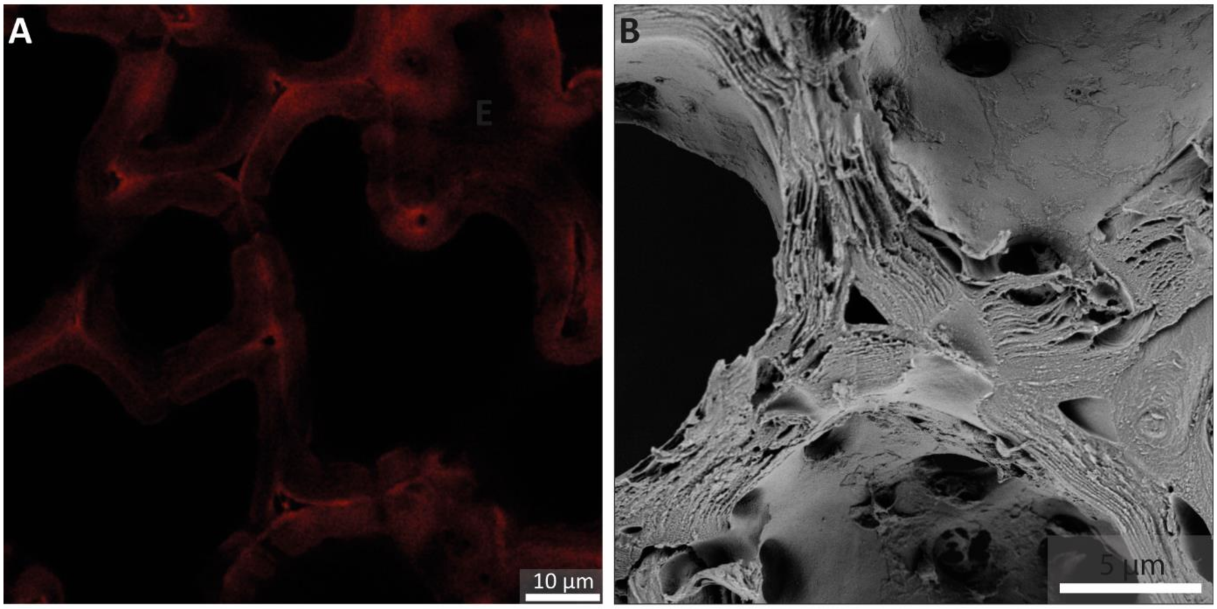

Supplementary Figure 5. Confocal fluorescence image and Scanning electron microscope (SEM) image of walnut shell inner part from October. (A) Confocal fluorescence image of lignin autofluorescence. (B) SEM image of inner part of walnut shell with substance stick to the cell wall.
